# Adsorption of Rare Earth Ce^3+^ and Pr^3+^ Ions by Hydrophobic Ionic Liquid

**DOI:** 10.1155/2021/6612500

**Published:** 2021-05-29

**Authors:** R. Devanathan, G. L. Balaji, R. Lakshmipathy

**Affiliations:** ^1^Department of Chemistry, Thanthai Hans Roever College, Perambalur 621212, Tamilnadu, India; ^2^School of Advanced Science and Languages, VIT Bhopal University, Bhopal 466114, Madhya Pradesh, India; ^3^Department of Chemistry, KCG College of Technology, Karapakkam, Chennai 600097, Tamilnadu, India

## Abstract

This study reports the use of hydrophobic ionic liquid (IL) based on D-galactose for the recovery of Ce (III) and Pr (III) ions from solutions. The equilibrium data were obtained by optimization of batch parameters, and various isotherms and kinetic models were utilised to predict the mechanistic process of sequestration of ions. The Arrhenius activation energies are found to be between 5–40 kJ, suggesting the physisorption process of ions onto IL. The present process is understood to be rapid and exothermic in nature according to thermodynamic experiments. The loading capacity was found to be 179.3 g L^−1^ and 141.5 g L^−1^, respectively, for Ce (III) and Pr (III) ions at pH 5 with a contact time of 30 min and dose being 0.1 g L^−1^. The higher uptake capacity is attributed to the presence of a highly electronegative fluorine atom in the IL. These results highlight the potential application of IL in the sequestration of Ce (III) and Pr (III) ions from any water sources.

## 1. Introduction

Rare earth elements (REEs) are found to have imperative applications in high-tech consumer products such as television, cell phones, and many other electronic appliances. REEs find wider applications in mundane and futuristic domains because of their chemical, electrical, optical and magnetic values [1]. A burgeoning population, modernization, and rapid industrialization have created a huge demand for the elements that are less commonly exploitable due to geochemical properties and dispersion [2]. With the ever-growing demand, the recovery of RREs from wastewater streams will be highly desirable and economical rather than depending on the mineral ore deposits.

Several techniques for the recovery and preconcentration of RREs are in practice such as coprecipitation [3], ion exchange [4], solvent extraction [5], and adsorption [6]. All the abovementioned techniques except adsorption find limitations due to the high cost of the operation, production of pollutants, and low recovery rates. Adsorption is a premier technique found to be a powerful and cost-effective method for the elimination and recovery of heavy metal ions and precious RREs. Many adsorbents such as activated carbon [6], agricultural waste and by-products [7], and organic frameworks [8] are found to be prolific in terms of cost and being ecofriendly; however, the efficiency and regeneration is always a concerning factor. Recently, the development of nanomaterials and ionic liquids has led to a new era of technological advancements in the field of wastewater treatments and recovery of REEs. Gul et al. published an interesting review on carbon-based nanoadsorbents for advanced water treatment [9]. Dhiman and Kondal reported the review of popular ZnO nanoadsorbents for the effective treatment of wastewater which highlights the role of ZnO nanoadsorbents [10]. In spite of the high efficiency exhibited by nanomaterials, the cost of production and unknown toxicity remain less prolific.

Ionic liquids are a class of salts that commonly contains short-lived species and ions and has exhibited enhanced distribution coefficients for heavy metal ions abolition [11, 12]. However, the liquid-phase ionic liquids require chelating agents to extract the cations from liquid phases [13]. Ionic liquids anchored onto nanomaterials were also explored for the extraction of cations from liquid phases [14, 15]. Hydrophobic ionic liquids have exhibited high efficiency towards cation removal and have put an end to choosing a chelating agent or support material [16]. Hydrophobic ionic liquids have exhibited greater efficiency towards the removal of cations from liquid phases [17]. Ionic liquids have been used in the removal and recovery of rare earth elements. Sol-gel materials doped with ionic liquids have been explored for the elimination of Ce (III) ions in liquid phases [18]. The application of hydrophobic ionic liquids in the removal and storing of rare earth elements are yet to be explored fully. In continuation to the earlier reports, the present investigation aims at the removal of rare earth elements such as trivalent Ce and Pr ions from liquid phases by hydrophobic PF_6_-anion-based ionic liquid.

## 2. Materials and Methods

**Scheme 1:**
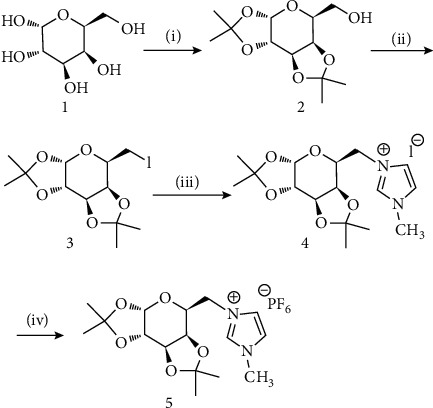
Reactants and setup: (i) dry CH_3_COCH_3_, zinc chloride, sulphuric acid (cat.), RT (97%); (ii) iodine, PPh_3_, imidazole, C_6_H_6_CH_3_, 80 oC, 3 h (82%); (iii) NMI, acetonitrile, reflux, six days (89%); and (iv) NaPF_6_, H_2_O (80%).

The synthesis of galactose-based IL (V) was carried out as per the earlier reports [19]. The scheme of the synthesis of IL5 is represented in Scheme 1. The white solid obtained at the final step was washed with water several times under vacuum.

### 2.1. Batch Adsorption Studies

In order to optimize the parameters for the removal of Ce (III) and Pr (III) ions, batch mode adsorption was adopted. Variables such as H^+^ concentration, time of contact, IL5 dose, and preliminary rare earth metal ion concentration were optimized by varying each parameter and keeping the remaining variables unchanged. The hydronium ion concentration of the solution was honed by changing the hydronium ion concentration from pH 2–9. The contact time between the IL5 and Ce (III) and Pr (III) ions was varied from 5 to 30 min, and the IL5 dosage was considered from 10 to 50 mg. The preliminary REE's ion amount was considered between 10 and 60 mg L^−1^. In addition to that mentioned above, the influence of temperature was also investigated by changing the variable from 303 to 323 K. The temperature-controlled orbital shaker was equipped to carry out all the experiments, and the test solutions were agitated at 150 rpm in a 50 ml conical flask. After each parameter optimization, the solid was recovered by centrifugation and the residual remaining was analysed with flame-AAS to quantify the residual concentration of the trivalent Ce and Pr ions. The percentage removal and maximum sequestration capacity of the IL5 were found by the following expressions:

(1) $$\begin{array}{c} \%\text{ removal}=\frac{C_{i}-C_{f}}{C_{i}}\text{x}100,\\ q_{e}=\left(C_{i}-C_{f}\right)\frac{V}{M}. \end{array}$$

## 3. Results and Discussion

The synthesized IL5 was evaluated as a potential sorbent for the sequestration of Ce (III) and Pr (III) ions from liquid phases by the batch process.

### 3.1. Effect of H^+^ Ion Concentration

**Figure 1:**
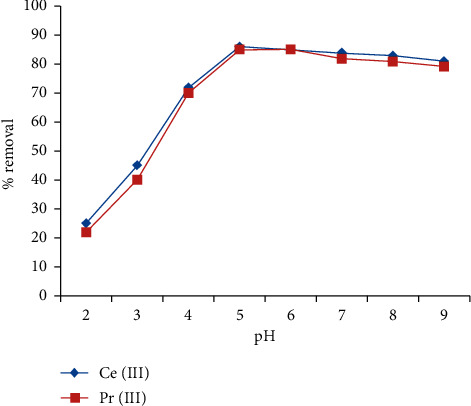
Effect of pH on the removal of Ce (III) and Pr (III) ions from the aqueous solution by IL.

The H^+^ ion concentration is one of the imperative parameters that must be optimized prior to any adsorption studies. The H^+^ ions' concentration in the solution plays a vital role in the binding of cations onto the surface of the adsorbent. In order to fix the H^+^ ion concentration of the solution, investigations were performed on changing the hydronium ion concentration from 2–9, and the results are shown in Figure 1. It is observed that, with increasing pH, the sequestration capacity of the IL5 increased for both the cations and was found to be maximum at pH 5. The surge in elimination ability is because of reducing amounts of H^+^ ion that competes with the trivalent Ce and Pr ions for the active sites on IL5. Beyond pH 5 for Ce^3+^ and pH 6 for Pr^3+^, the removal efficiency from the aqueous solution was found to decrease, and this may be due to the surface of IL5 being positive and the formation of several hydroxides. Moving forward, pH 5 was set as the optimal H^+^ concentration for later investigations.

### 3.2. Influence of IL5 Dosage

**Figure 2:**
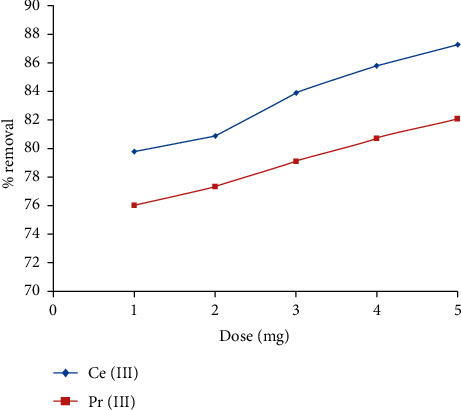
Influence of IL dose on the elimination of Ce (III) and Pr (III) ions from the aqueous solution.

The quantity of the sorbent required is of significance in selecting an adsorbent for any adsorption system, and any classic adsorbent displays high output at low dosage levels. In order to optimize and evaluate the efficacy of the IL5 dose, the dose was varied from 0.1 to 0.5 g L^−1^, and the outputs are represented in Figure 2. The results clearly depict that, at a very low dosage, the IL5 exhibited over 75% of removal efficiency. Furthermore, with the surge in dosage, the ability of removal increases, but the efficacy is substantially small and negligible. The high removal efficiency of IL5 at low dose levels indicates that IL5 is a prolific sorbent for the elimination of Ce (III) and Pr (III) ions from liquid phases.

### 3.3. Influence of Contact Time

**Figure 3:**
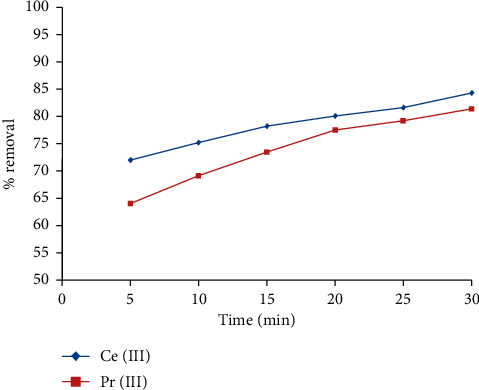
Influence of time of contact on the removal of Ce (III) and Pr (III) ions from the aqueous solution by IL.

Investigations were performed on varying the contact time (5 to 30 min) to fix the optimum time of contact for the sorption of trivalent Ce and Pr ions from liquid phases, and the outputs are represented in Figure 3. The adsorption of Ce (III) and Pr (III) ions was rapid within 5 min of time of contact and gradually increased with the extension of time. The swift removal efficiency noticed within 5 min of time must be due to spontaneous occupation of active sites, and slower adsorption with burgeoning time is due to exhaustion of sites on the plane of IL5. Moving forward, 30 min of time was fixed as the optimum time for other investigations.

### 3.4. Desorption of Ce (III) and Pr (III) Ions from IL5

**Figure 4:**
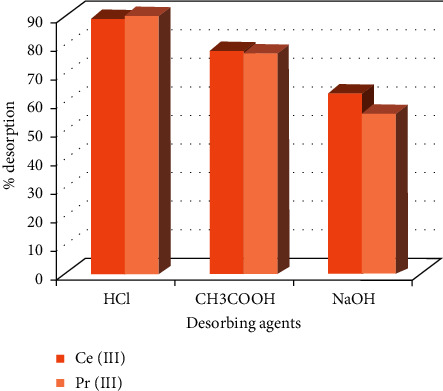
Desorption efficiency exhibited by various desorbing agents on Ce (III) and Pr (III) ions.

Desorption and regeneration of the adsorbent are of prime importance since the desorbed cations can be recovered and used for further application. The regenerated adsorbent can be reused again in the sorption process which makes the process economical. In view of that mentioned above, the adsorbed Ce (III) and Pr (III) ions onto IL5 were desorbed by contacting with various desorbing agents such as 0.01 M HCl, 0.01 M CH_3_COOH, and 0.01 M NaOH, and Figure 4 represents the outcomes. It is evident that the 0.01 M HCl was superior in desorbing the Ce (III) and Pr (III) ions from IL5 followed by 0.01 M CH_3_COOH and NaOH. The regenerated IL5 was reused for two successive cycles.

### 3.5. Adsorption Isotherms

At equilibrium, the initial concentration of the trivalent Ce and Pr ions was varied to estimate the uptake capacity of the present ionic liquid. The initial quantities of selected ions were changed from 10 to 60 mg L^−1^, and the uptake ability was calculated to be 179.3 and 141.5 g L^−1^ for Ce (III) and Pr (III) ions, respectively. The superior electronegative atom such as fluorine present in the anion part of IL is primarily responsible for supreme loading capacity exhibited by IL5. The electron cloud around the 6 F^−^ atoms attracts the positively charged Ce (III) and Pr (III) ions. In order to predict the mechanistic process of adsorption, two well-known mathematical models such as Langmuir and Freundlich adsorption isotherms were employed.

**Figure 5:**
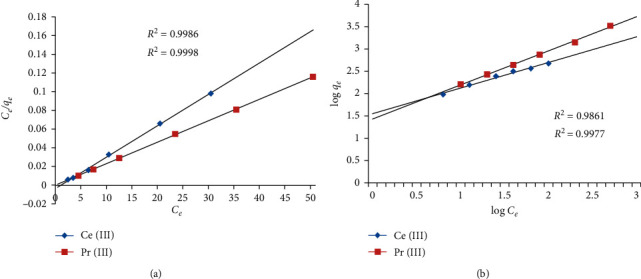
Isotherm plots of (a) Langmuir and (b) Freundlich for the elimination of Ce (III) and Pr (III) ions from liquid phases by IL.

The plots of selected isotherms models as mentioned above are shown in Figures 5(a) and 5(b), and the respective constants and coefficient of correlation are tabulated in Table 1. The coefficients of correlation for Langmuir and Freundlich isotherms were observed to be close to one recommending the applicability of the models for the recovery of Ce (III) and Pr (III) ions from liquid phases by IL5. Furthermore, the relevance of the Langmuir model is backed up by the theoretical loading capacity that is the same as the experimental values. The binding of Ce (III) and Pr (III) ions onto IL5 is a multilayer adsorption process, and each layer obeys Langmuir isotherm.

### 3.6. Adsorption Kinetics

Kinetic investigations provide valuable information on the mechanism of removal of the analytes, and experiments were performed in line with the equilibrium studies for Ce (III) and Pr (III) ions at various preliminary REE ion concentration and temperatures. The residual concentrations of test samples obtained at preset time intervals were analysed for understanding the mechanistic aspects of adsorption. Various kinetic models helpful in predicting the mechanism of binding were adopted, and the obtained kinetic data were analysed.

The coefficients of correlation and respective constants of the models used for understanding the mechanism of binding of Ce (III) and Pr (III) ions onto IL5 are summarized in Tables 2 and 3. The coefficients of correlation at different temperatures and concentrations obtained for pseudo-second-order were found to be high compared to those of the other two models used in this study. The correlation coefficients of the pseudo-second-order kinetic model are very near to one, and the loading capacity theoretical values are very similar to experimental advising the better fit of the model. These observations suggest that the binding of Ce (III) and Pr (III) ions onto IL5 is governed by chemical reactions. However, for an adsorption process to be controlled by chemical reactions, many setups must be satisfied. These conditions are as follows [20]:

- The constants of the rate of reaction should remain unchanged irrespective of the initial concentration of target ions
- The size of the adsorbent particle should not alter the rate constant
- The degree of shaking should not influence the rate constant

If any of the abovementioned conditions are not obeyed, then rate controlling is not chemical reaction kinetics even though the kinetic values follow the pseudo-second-order model [13]. Experiments were carried out at two different initial metal ion concentrations of Ce (III) and Pr (III) ions onto IL5 in order to check whether the rate of reaction is controlled by chemical reactions or not. In Tables 2 and 3, the rate constant *k* appears to be not inconsistent for varying initial metal ion concentration of Ce (III) and Pr (III) ions onto IL5. The inconsistency in the value of *k* for varying initial concentration of counter ions suggests that chemical reactions are not the rate-limiting step, even though kinetic data tend to fit well with the pseudo-second-order model. Communications observed were similar to this study for the elimination of Cd^2+^ ions by ionic liquid [17] and the removal of Pb^2+^ and Cu^2+^ ions by Watermelon rind [7].

The Arrhenius equation expresses temperature as a function for the second-order rate constant.

(2) $$\begin{array}{c} \ln\;k_{2}=\ln\;A-\frac{E_{a}}{\text{RT}}. \end{array}$$

The Arrhenius activation energy calculated for the varying preliminary metal ion concentrations of Ce (III) and Pr (III) is tabulated in Table 4. Interestingly, the activation energy for both metal ions at two different initial concentrations is less than 40 kJ, suggesting that the process is physisorption. In general, chemisorption has higher activation energy between 40 and 800 kJ/mol. These observations conclude that the current sorption system is physisorption.

Furthermore, to understand the diffusion mechanism, the rate data were analysed with the intraparticle diffusion model.

(3) $$\begin{array}{c} q_{t}=k_{\mathrm{int}}t^{1/2}+C. \end{array}$$

If the resulting plot is linear, it suggests that the process is completely controlled by intraparticle diffusion, and the multilinear plot indicates two or more steps of a diffusion process. In the present process, the recovery of Ce (III) and Pr (III) ions by IL5 is intraparticle diffusion only and it is concluded by the liner plots obtained from the experimental results.

### 3.7. Thermodynamics of Adsorption

The removal of Ce (III) and Pr (III) ions by IL5 was investigated at varying temperatures in order to understand the nature of the adsorption process. It was found that the efficiency of removal and loading capacity decreased with the surge in temperature and this must be because of the weakening of the surface binding spots of IL5 that binds the Ce (III) and Pr (III) ions. Also, with the surge in temperature, the kinetic energy of the analytes increases, and as a result, the probability of counterions meeting the active sites of IL5 decreases. Furthermore, the thermodynamic parameters which reveal the nature of the adsorption process were derived and summarized in Table 5.

(4) $$\begin{array}{c} K_{D}=\frac{q_{e}}{C_{e}},\\ \Delta G^{{}^{\circ}}=-\text{RT}\ln\;K_{D},\\ \Delta G^{{}^{\circ}}=\Delta H^{{}^{\circ}}-T\Delta S^{{}^{\circ}}. \end{array}$$

The variables of thermodynamics such as Gibbs free-energy change (∆*G*°), enthalpy (∆*H*°), and entropy (∆*S*°) derived for the present process are tabulated in Table 5. The free-energy change (∆*G*°) at all temperatures is found to be negative for the recovery of target analyte ions from an aqueous solution by IL5, suggesting that the process is spontaneous. However, with the rise in temperature, the negative values of ∆*G*° were found to decline, suggesting that the spontaneity decreases with the rise in temperature. The lowering of loading capacity also supports this observation. The exothermic nature of the present process was understood from the negative values of enthalpy (∆*H*°), and the positive values of entropy (∆*S*°) highlight that the entropy increases at the interface of Ce (III) and Pr (III) ions and IL5.

### 3.8. Influence of Salt Ionic Strength

It is salient to consider the influence of ionic strength of various salts on the removal and recovery of Ce (III) and Pr (III) ions from aqueous solution. In general, effluents tend to contain various ions usually due to salts and hardness of water, and in order to understand the influence of those, efforts were made to understand their role. Sorption experiments were conducted in the presence of varying salt concentrations for the removal and recovery of Ce (III) and Pr (III) ions from the aqueous solution, and the results are tabulated in Table 6. The results were found to be interesting as seen in Table 6. The presence of Na^+^ ions has a partial impact on the loading capacity of Ce (III) and Pr (III) ions, and this is due to Na^+^ ions being more electropositive than the target ions. However, the presence of Ca^2+^ and Mg^2+^ ions has not influenced the loading capacity, and this is due to the electronegativity of the Ca^2+^ and Mg^2+^ ions being the same and greater than the Ce (III) and Pr (III) ions. These results suggest that the hardness of water does not have any influence on loading capacity, but the presence of Na^+^ ions must be considered prior to the sorption of Ce (III) and Pr (III) ions from the aqueous solution.

### 3.9. Adsorption Mechanism

The mechanism of removal of Ce (III) and Pr (III) ions by IL is attributed to the electrostatic attraction of the cations on the highly electronegative fluorine atoms present in the form of PF_6_^−^ ions on IL. The fluorine atoms on the IL form an electron cloud over them due to their high electronegativity, and this could attract the positively charged cations electrostatically. The electrostatic attraction mechanism is supported by variation of hydronium ion concentration where the high concentration of H^+^ ions resulted in the low attraction of Ce (III) and Pr (III) ions, while at lower H^+^ ion concentration, the sorption was found to be superior. The salt ionic strength also corroborates the observations suggesting that the electrostatic attraction is the mechanism and physiosorption is taking place. Desorption and regeneration studies further support the physiosorption process and electrostatic attraction mechanism.

## 4. Conclusions

This study reported the potential application of carbohydrate-based hydrophobic ionic liquid with PF_6_^−^ ion as a prolific sequester for the recovery of Ce (III) and Pr (III) ions from aqueous solution. Adsorption batch investigations were performed for optimizing the imperative variables such as pH, time of contact, the dosage of IL, and preliminary Ce (III) and Pr (III) ion concentrations. Adsorption isotherm investigations suggested that the present process is multilayer adsorption and each layer obeys Langmuir isotherm. Profound kinetic studies suggested that the process is physiosorption following a pseudo-second-order kinetic model. The physiosorption is also assisted by the Arrhenius activation energy calculated from various temperatures. The thermodynamic studies highlight the spontaneous and exothermic process of the present system. The results conclude that the present ionic liquid is a potential precursor for the recovery of Ce (III) and Pr (III) ions from water sources.

## Figures and Tables

**Table 1:** Constants of Freundlich and Langmuir isotherm derived for the recovery of Ce (III) and Pr (III) ions from liquid phases by IL (temperature −303 K and dose 0.5 mg g^−1^).

Isotherm	Parameters	Ce (III)	Pr (III)
Freundlich	*K*_f_ (mg g^−1^)	6.23	8.91
	1/*n* (L mg^−1^)	0.021	0.017
	*R* ^2^	0.975	0.979

Langmuir	*V*_m_ (mg g^−1^)	169.2	144.4
	*b* (L mg^−1^)	0.023	0.031
	*R* ^2^	0.990	0.981

**Table 2:** Kinetic constants of Ce (III) ions adsorption at various concentrations of Ce (III) ion and temperature (dose- 0.5 mg g^−1^).

Kinetic model	Parameters	303 K		313 K		323 K	
		10 (mg L^−1^)	30 (mg L^−1^)	10 (mg L^−1^)	30 (mg L^−1^)	10 (mg L^−1^)	30 (mg L^−1^)
Experimental	*q*_e_ (mg g^−1^)	53.5	126.3	52.9	125.8	51.7	123.9

Pseudo-first-order	*k*_1_ (min^−1^)	0.023	0.096	0.033	0.109	0.037	0.122
	*q*_e_ (mg g^−1^)	12.4	21.1	11.7	20.7	10.5	19.7
	*R* ^2^	0.890	0.863	0.904	0.853	0.909	0.899

Pseudo-second-order	*k*_2_ (g mg^−1^min^−1^)	0.009	0.007	0.010	0.008	0.012	0.010
	*q*_e_ (mg g^−1^)	54.1	126.8	53.8	126.2	53.2	125.7
	*R* ^2^	0.999	0.999	0.999	0.999	0.999	0.999

Elovich	*α* (mg g^−1^ min^−1^)	2.1 × 10^−25^	2.0 × 10^−26^	2.3 × 10^−26^	2.1 × 10^−27^	2.4 × 10^−27^	2.3 × 10^−27^
	*β* (mg g^−1^)	1.16	1.87	1.24	1.85	1.33	1.88
	*R* ^2^	0.765	0.754	0.806	0.813	0.854	0.833

**Table 3:** Kinetic constants of Pr (III) ions adsorption by IL at various preliminary Pr (III) ion concentrations and temperature (dose- 0.5 mg g^−1^).

Kinetic model	Parameters	303 K		313 K		323 K	
		10 (mg L^−1^)	30 (mg L^−1^)	10 (mg L^−1^)	30 (mg L^−1^)	10 (mg L^−1^)	30 (mg L^−1^)
Exploratory	*q*_e_ (mg g^−1^)	47.1	107.7	46.7	106.6	46.1	106.1

Pseudo-first-order	*k*_1_ (min^−1^)	0.027	0.117	0.025	0.113	0.022	0.109
	*q*_e_ (mg g^−1^)	11.6	21.3	11.2	20.6	10.7	19.8
	*R* ^2^	0.911	0.903	0.908	0.897	0.898	0.889

Pseudo-second-order	*k*_2_ (g mg^−1^ min^−1^)	0.016	0.011	0.017	0.013	0.019	0.015
	*q*_e_ (mg g^−1^)	46.3	106.9	45.7	106.5	44.8	105.8
	*R* ^2^	0.999	0.998	0.999	0.999	0.999	0.999

Elovich	*α* (mg g^−1^ min^−1^)	1.9 × 10^−26^	2.1 × 10^−26^	2.3 × 10^−26^	2.2 × 10^−26^	2.5 × 10^−26^	2.7 × 10^−26^
	*β* (mg g^−1^)	1.06	1.24	1.13	1.28	1.20	1.37
	*R* ^2^	0.802	0.821	0.843	0.818	0.876	0.813

**Table 4:** Arrhenius activation energy for the elimination of trivalent Ce and Pr ions from the aqueous phase by IL.

Ion	Concentration (mg L^−1^)	*E*_a_ (kJ mol^−1^)
Ce (III)	10	15.7
	30	26.9

Pr (III)	10	16.4
	30	29.1

**Table 5:** Thermodynamic variables calculated for the recovery of Ce (III) and Pr (III) ions by IL (time- 30 min, dose- 0.5 mg g^−1^).

Metal ion	Temperature (K)	Sorption capacity (*q*_e_ mg g^−1^)	∆*G*° (kJ mol^−1^)	∆*H*° (kJ mol^−1^)	∆*S*° (J mol^−1^ k^−1^)
Ce (III)	303	53.5	−4.56	−5.22	692.3
	313	52.9	−4.21		
	323	51.7	−3.90		

Pr (III)	303	47.1	−4.12	−4.76	789.1
	313	46.7	−3.97		
	323	46.1	−3.66		

**Table 6:** Influence of salt ionic strength on the elimination of Ce (III) and Pr (III) ions from the solution (time- 30 min, dose- 0.5 mg g^−1^, and temperature- 303 K).

Ionic strength (mol L^−1^)	Salt	Sorption capacity (mg g^−1^)	
		Ce (III)	Pr (III)
0	—	53.5	47.1

0.01	NaCl_2_	51.9	46.2
	CaCl_2_	53.2	46.9
	MgCl_2_	53.5	47.2

0.1	NaCl_2_	49.3	43.4
	CaCl_2_	52.3	46.2
	MgCl_2_	53.1	46.8

## Data Availability

The data of this study are available in the corresponding author's Google Sites page.
